# Discriminative Structure Learning of Bayesian Network Classifiers from Training Dataset and Testing Instance

**DOI:** 10.3390/e21050489

**Published:** 2019-05-13

**Authors:** Limin Wang, Yang Liu, Musa Mammadov, Minghui Sun, Sikai Qi

**Affiliations:** 1 Key Laboratory of Symbolic Computation and Knowledge Engineering of Ministry of Education, Jilin University, Changchun 130012, China; 2 College of Computer Science and Technology, Jilin University, Changchun 130012, China; 3 Faculty of Science, Engineering & Built Environment, Deakin University, Burwood, VIC 3125, Australia

**Keywords:** Bayesian network classifiers, Markov blanket, target learning

## Abstract

Over recent decades, the rapid growth in data makes ever more urgent the quest for highly scalable Bayesian networks that have better classification performance and expressivity (that is, capacity to respectively describe dependence relationships between attributes in different situations). To reduce the search space of possible attribute orders, *k*-dependence Bayesian classifier (KDB) simply applies mutual information to sort attributes. This sorting strategy is very efficient but it neglects the conditional dependencies between attributes and is sub-optimal. In this paper, we propose a novel sorting strategy and extend KDB from a single restricted network to unrestricted ensemble networks, i.e., unrestricted Bayesian classifier (UKDB), in terms of Markov blanket analysis and target learning. Target learning is a framework that takes each unlabeled testing instance P as a target and builds a specific Bayesian model Bayesian network classifiers (BNC)${}_{P}$ to complement BNC${}_{T}$ learned from training data T. UKDB respectively introduced UKDB${}_{P}$ and UKDB${}_{T}$ to flexibly describe the change in dependence relationships for different testing instances and the robust dependence relationships implicated in training data. They both use UKDB as the base classifier by applying the same learning strategy while modeling different parts of the data space, thus they are complementary in nature. The extensive experimental results on the Wisconsin breast cancer database for case study and other 10 datasets by involving classifiers with different structure complexities, such as Naive Bayes (0-dependence), Tree augmented Naive Bayes (1-dependence) and KDB (arbitrary *k*-dependence), prove the effectiveness and robustness of the proposed approach.

## 1. Introduction

Since 1995, researchers have proposed to embed machine-learning techniques into a computer-aided system, such as medical diagnosis system [1,2,3,4]. Andres et al. [5] proposed an ensemble of fuzzy system and evolutionary algorithm for breast cancer diagnosis, which can evaluate the confidence level to which the system responds and clarifies the working mechanism of how it derives its outputs. Huang et al. [6] constructed a hybrid SVM-based strategy with feature selection to find the important risk factor for breast cancer. Generally speaking, without domain-specific expertise in medicine, researchers in data mining prefer models with high classification accuracy and low computational complexity. In contrast, common people (including patients and their relatives) hope that the models can have high-level interpretability simultaneously. Bayesian network classifiers (BNCs) are such models that can graphically describe the conditional dependence between attributes (or variables) and be considered to be one of the most promising graph models [7,8]. It can mine statistical knowledge from data and infer under conditions of uncertainty [9,10]. BNCs, from 0-dependence Naive Bayes (NB) [11] to 1-dependence tree augmented Naive Bayes (TAN) [12], then to arbitrary *k*-dependence Bayesian classifier (KDB) [13], can represent the knowledge with complex or simple network structure. KDB can theoretically represent conditional dependence relationships of arbitrary complexity. However, this approach is not effective for some specific cases. The model learned from training data may not definitely fit all testing instances. Otherwise, its bias and variance will always be 0, which is against the bias-variance dilemma [14]. In the case of breast cancer, for different specific cases, the dependence relationships between attributes may be different. For BNCs, conditional mutual information (CMI) [15], $I(X_{i};X_{j}|C)$, is commonly used to measure the conditional dependence relationship between attributes $X_{i}$ and $X_{j}$ given class variable *C*:

(1) $$\begin{array}{cc} I(X_{i};X_{j}|C) & =\sum_{x_{i}\in X_{i}}\sum_{x_{j}\in X_{j}}\sum_{c\in C}P\left(x_{i},x_{j},c\right)log\frac{P(x_{i},x_{j}|c)}{P\left(x_{i}|c\right)*P\left(x_{j}|c\right)}\\ & =\sum_{x_{i}\in X_{i}}\sum_{x_{j}\in X_{j}}\sum_{c\in C}I\left(x_{i};x_{j}|c\right). \end{array}$$

**Figure 1:**
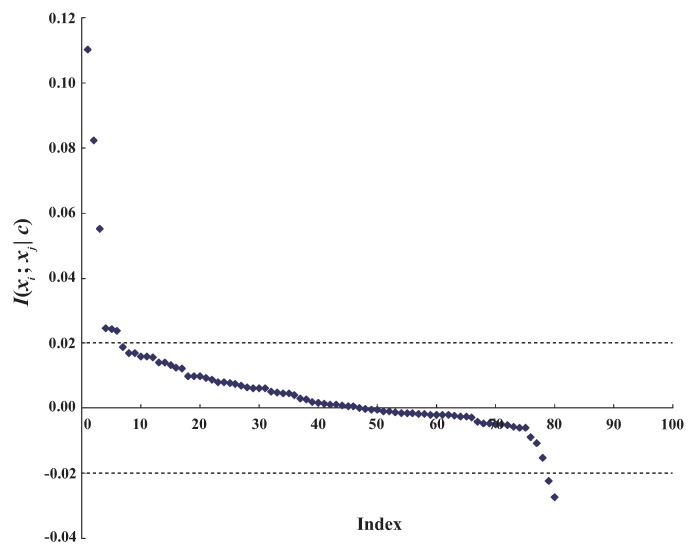
The distribution of $I(x_{i};x_{j}|c)$ between attributes $X_{1}$ and $X_{2}$ on dataset WBC.

$I(X_{i};X_{j}|C)$ can measure the conditional dependence between attributes between attributes $X_{i}$ and $X_{j}$ given class *C*. Correspondingly, $I(x_{i};x_{j}|c)$ can measure the conditional dependence between them when they take specific values. When $P\left(x_{i},x_{j}|c\right)>P\left(x_{i}|c\right)*P\left(x_{j}|c\right)$ or $log(P\left(x_{i},x_{j}|c\right)/\left(P\left(x_{i}|c\right)*P\left(x_{j}|c\right)\right)>0$, $I(x_{i};x_{j}|c)>0$ holds and the relationship between attribute values $x_{i}$ and $x_{j}$ can be considered to be conditional dependence. In contrast, when $P\left(x_{i},x_{j}|c\right)<P\left(x_{i}|c\right)*P\left(x_{j}|c\right)$ or $log(P\left(x_{i},x_{j}|c\right)/\left(P\left(x_{i}|c\right)*P\left(x_{j}|c\right)\right)<0$, $I(x_{i};x_{j}|c)<0$ holds and we argue that the relationship between attribute values $x_{i}$ and $x_{j}$ can be considered to be conditional independence. When $P\left(x_{i},x_{j}|c\right)=P\left(x_{i}|c\right)*P\left(x_{j}|c\right)$ and $I(x_{i};x_{j}|c)=0$, the relationship between attribute values $x_{i}$ and $x_{j}$ just turns from conditional dependence to conditional independence. On dataset WBC (breast cancer), $I(X_{1};X_{2}|C)$ achieves the largest value of CMI (0.4733) among all attribute pairs. The distribution of $I(x_{i};x_{j}|c)$, which correspond to different attribute value pairs of $X_{1}$ and $X_{2}$, are shown in Figure 1. As shown in Figure 1, the relationship between attributes $X_{1}$ and $X_{2}$ is dependent in general because the positive values of $I(x_{1};x_{2}|c)$, which represent conditional dependence, have a high proportion among all the values. In addition, some $I(x_{1};x_{2}|c)$ values are especially large. In contrast, there also exist some negative values of $I(x_{1};x_{2}|c)$ that represent conditional independence, i.e., the dependence relationship may be different rather than invariant when attributes take different values. However, general BNCs (like NB, TAN and KDB), which only build one model to fit training instances, cannot capture this difference and cannot represent the dependence relationships flexibly.

To meet the needs of experts in machine learning or in medicine, common people (including patients and their relatives) and the problem of breast cancer mentioned above, we propose a novel sorting strategy and extend KDB from a single restricted network to unrestricted ensemble networks, i.e., unrestricted *k*-dependence Bayesian classifier (UKDB), in terms of Markov blanket analysis and target learning. Target learning [16] is a framework that takes each unlabeled testing instance P as a target and builds a specific Bayesian model BNC${}_{P}$ to complement BNC${}_{T}$ learned from training data T.

To clarify the basic idea of UKDB, we introduce two concepts: “Domain knowledge”, which expresses a general knowledge framework learned from the training data, it focuses on describing interdependencies between attributes, such as attribute $A_{1}$ and $B_{1}$. In addition, “Personalized knowledge”, which expresses a specific knowledge framework learned from the attribute values in the testing instance, such as attribute $A_{1}=a_{1}$ and $B_{1}=b_{1}$. Take breast cancer as an example, there is a strong correlation between attributes “Clump Thickness” and “Uniformity of Cell Size” (corresponding CMI achieves the maximum value, i.e., 0.4733), which can be considered to be the domain knowledge. In contrast, for a testing instance with attribute values “Clump Thickness = 1” and “Uniformity of Cell Size = 3”, the dependence relationship between those attribute values is approximately independent (corresponding value of CMI is 0.0002), which can be regarded as the personalized knowledge. The personalized knowledge with clear expressivity (capacity to respectively describe dependence relationships between attributes in different situations.) and tight coupling (capacity to describe the most significant dependencies between attributes.) makes ever more urgent the quest for highly scalable learners.

UKDB contains two sub-models: UKDB${}_{T}$ and UKDB${}_{P}$. UKDB${}_{T}$ is learned from training data T, which can be thought of as a spectrum of dependencies and is a statistical form of domain knowledge. UKDB${}_{P}$ is a specific BNC to mine the personalized knowledge implicated in each single testing instance P, i.e., the specific knowledge that describes the conditional dependency between the attribute values in each single testing instance P. UKDB${}_{P}$ and UKDB${}_{T}$ apply the same strategy to build the network structure, but they apply different probability distributions and target different data spaces, thus they are complementary in nature, i.e., in contrast to restricted BNC, e.g., KDB, UKDB can discriminatively learn different unrestricted Bayesian network structures to represent different knowledge from training dataset and testing instance, respectively.

The Wisconsin breast cancer (WBC) database [17] is usually used as a benchmark dataset [1,2,3,4] and is also selected in our main experiments for case study to demonstrate personalized Bayesian networks (BN) structures. The case study on the WBC database, as well as an extensive experimental comparison on additional 10 UCI datasets by involving some benchmark BNCs, show the advantages of the proposed approach.

## 2. Bayesian Network and Markov Blanket

All the symbols used in this paper are shown in Table 1. We wish to build a Bayesian network classifier from labeled training dataset T such that the classifier can estimate the probability P(c|x) and assign a discrete class label $c\in \Omega_{C}$ to a testing instance $x=(x_{1},\cdots ,x_{n})$. BNs are powerful tools for knowledge representation and inference under conditions of uncertainty. A BN consists of two parts: the qualitative one in the form of a directed acyclic graph. Each node of the graph represents a variable in the training data and the directed edges between pairs of nodes represent dependence relationships between them; and the quantitative one based on local probability distributions for specifying the dependence relationships. Even though BNs can deal with continuous variables, we exclusively discuss BNs with discrete nodes in this paper. Directed edges represent statistical or causal dependencies among the variables. The directions are used to define the parent-children relationships. For example, given an edge X→Y, *X* is the parent node of *Y*, and *Y* is the children node.

A node is conditionally independent of every other node in the graph given its parents ($X_{p}$), its children ($X_{c}$), and the other parents of its children ($X_{cp}$). $\left\{X_{p},X_{c},X_{cp}\right\}$ forms the Markov blanket of the node [7], which contains all necessary information or knowledge to describe the relationships between that node and other nodes. BNCs are special type of BNs. By applying different learning strategies, BNCs encode the dependence relationships between predictive attributes $X=\{X_{1},\cdots ,X_{n}\}$ and class variable *C*. Thus, the Markov blanket for variable *C* can provide the necessary knowledge for classification.

Suppose that *X* is divided into three parts, i.e., $X=\{X_{p},X_{c},X_{cp}\}$, the joint probability distribution P(x,c) can be described in the form of chain rule,

(2) $$\begin{array}{cc} P(x,c) & =P(x_{p},x_{cp},x_{c},c)\\ & =P\left(x_{p}\right)P\left(c|x_{p}\right)P\left(x_{cp}|x_{p},c\right)P\left(x_{c}|x_{cp},x_{p},c\right) \end{array}$$

**Figure 2:**
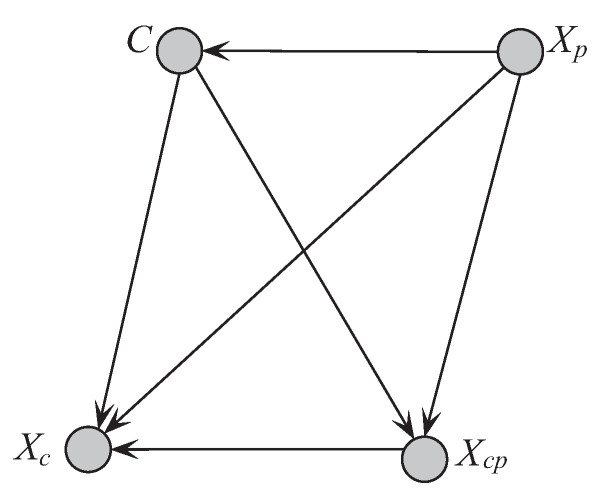
Unrestricted Bayesian classifier corresponding to joint probability distribution.

The unrestricted BNC shown in Figure 2, which corresponds to (2), is a full Bayesian classifier (i.e., no independencies). The computational complexity in such an unrestricted model is an NP-hard problem.

NB is the simplest of the BNCs. Given the class variable *C*, the predictive attributes are supposed to be conditionally independent of one another, i.e.,

(3) $$P_{\mathrm{NB}}\left(x|c\right)=\prod_{i=1}^{n}P\left(x_{i}|c\right).$$

**Figure 3:**
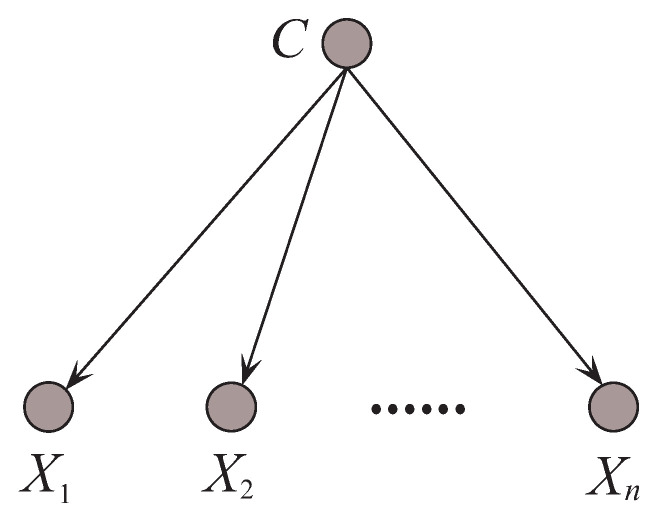
An example of Naive Bayes.

Even though the supposition rarely holds, its classification performance is competitive to some benchmark algorithms, e.g., decision tree, due to the insensitivity to the changes in training data and approximate estimation of the conditional probabilities $P(x_{i}|c)$ [10]. Figure 3 shows the structure of NB. In contrast to Figure 2, there exists no edge between attribute nodes for NB and thus it can represent 0 conditional dependencies. It is obvious that the conditional independence assumption is too strict to be true in reality. When dealing with complex attribute dependencies, that will result in classification bias.

**Figure 4:**
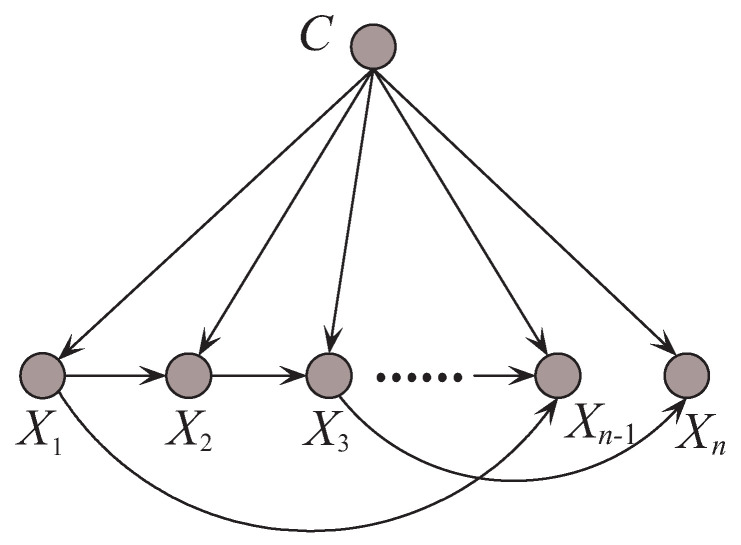
An example of Tree augmented Naive Bayes.

TAN relaxes the independence assumption and extends NB from 0-dependence tree to 1-dependence maximum weighted spanning tree [12]. The joint probability for TAN turns to be

(4) $$P_{\mathrm{TAN}}\left(x,c\right)=P\left(c\right)P\left(x_{1}|c\right)\prod_{i=2}^{n}P\left(x_{i}|c,x_{j}\right),$$

where $X_{j}$ is the parent attribute of $X_{i}$. The constraint on the number of parents intensively requires that only the most significant, i.e., 0+1+⋯+1=n−1, conditional dependencies are allowed to be represented. By comparing CMI, the edge between $X_{i}$ and $X_{j}$ will be added to the network in turn to build a maximal spanning tree. Once the conditional independence assumption does not hold, TAN is supposed to achieve better classification performance than NB. An example of TAN is shown in Figure 4.

KDB can represent arbitrary degree of dependence and control its bias/variance trade-off with a single parameter, *k*. By comparing mutual information (MI) $I(X_{i};C)$ [15], attributes will be sorted in descending order and enter the network structure in turn.

(5) $$I\left(X_{i};C\right)=\sum_{x_{i}\in X_{i}}\sum_{c\in C}P\left(x_{i},c\right)log\frac{P(x_{i},c)}{P\left(x_{i}\right)P\left(c\right)}$$

To control the structure complexity, each attribute $X_{i}$ is required to have no more than *k* parent attributes. Thus, for any of the first k+1 attributes in the order, they will indiscriminately select all the attributes already in the model as its parents. For the other attributes, they will select *k* parent attributes which correspond to the highest values of $I(X_{i};X_{j}|C)$ where $X_{j}$ ranks before $X_{i}$.

**Figure 5:**
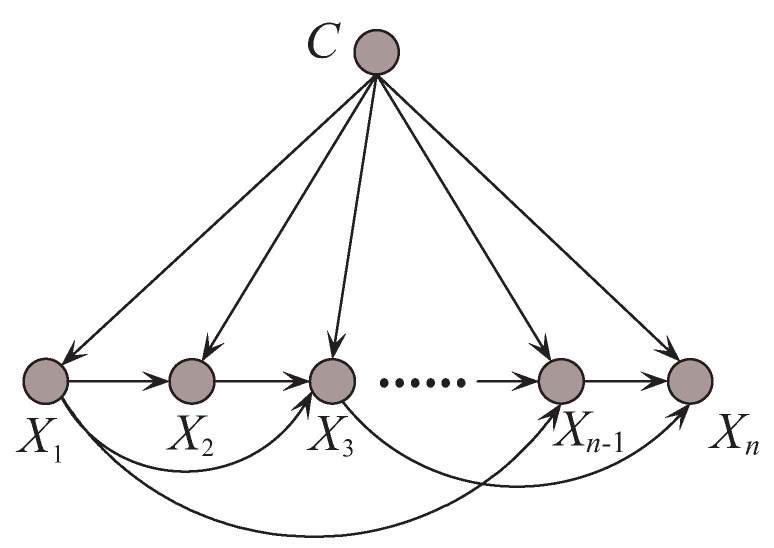
An example of *k*-dependence Bayesian classifier when *k* = 2.

Suppose that the attribute order is $\left\{X_{1},\cdots ,X_{n}\right\}$, the joint probability for KDB turns to be

(6) $$P_{\mathrm{KDB}}\left(x,c\right)=P\left(c\right)\prod_{i=1}^{n}P\left(x_{i}|c,\pi_{x_{i}}\right)$$

where $\pi_{x_{i}}=\left\{X_{i_{1}},\cdots ,X_{i_{j}}\right\}$ are the *j* parent attributes of $X_{i}$ in the structure, where j=min{i−1,k}. KDB can represent $nk-\frac{k^{2}}{2}-\frac{k}{2}$ conditional dependencies. When k=1, KDB represents the same number of conditional dependencies of TAN. As *k* increases, KDB can represent increasingly conditional dependencies. Figure 5 shows an example of KDB when *k* = 2.

Since KDB can be extended to describe dependence relationships of arbitrary degree and thus demonstrates its flexibility, researchers proposed many important refinements to improve its performance [18,19,20,21]. Pernkopf and Bilmes [22] proposed a greedy heuristic strategy to determine the attribute order by comparing $I(C;X_{i}|X_{j})$ where $X_{j}$ ranks higher than $X_{i}$ in the order, i.e., i>j. Taheri et al. [23] proposed to build a dynamic structure without specifying *k* a priori, and they proved that the resulting BNC is optimal.

## 3. The UKDB Algorithm

According to generative approach, the restricted BNCs, which take class variable *C* as the common parent of all predictive attributes, define a unique joint probability distribution P(x,c) in the form of chain rule of lower-order conditional probabilities,

(7) $$P\left(x,c\right)=P\left(c\right)P\left(x_{1}|c\right)P\left(x_{2}|x_{1},c\right)\cdots P\left(x_{n}|x_{1},\cdots ,x_{n-1},c\right).$$

The corresponding classification rule is

(8) $$\begin{array}{c} c^{*}=\arg\max P\left(x,c\right)=\arg\max P\left(c\right)P\left(x_{1}|c\right)\cdots P\left(x_{n}|x_{1},\cdots ,x_{n-1},c\right). \end{array}$$

To maximize P(x,c), an ideal condition is that each factor $P(x_{i}|x_{1},\cdots ,x_{i-1},c)$ will be maximized. In other words, $X_{i}$ should be strongly dependent on its parents, especially on class variable *C*. Given limited number of training instances, the reliability of conditional probability estimation $P(x_{i}|\Pi_{i},c)$ will increase as the dependence relationships between $X_{i}$ and its parent attributes increases. To achieve the trade-off between classification performance and structure complexity, only limited number of dependence relationships will be represented by BNs, e.g., KDB. In addition, the classification rule for KDB turns to be

(9) $$\begin{array}{c} c^{*}=\arg\max\hat{P}\left(x,c\right)=\arg\max P\left(c\right)\prod_{i=1}^{n}P\left(x_{i}|\Pi_{i},c\right), \end{array}$$

where $\Pi_{i}$ is one subset of $\left\{X_{1},\cdots ,X_{i-1}\right\}$ and contains at most *k* attributes. Obviously, $P\left(x,c\right)\neq \hat{P}\left(x,c\right)$. No matter what the attribute order is, the full BNC represents the same joint distribution, i.e., P(x,c). In contrast, from Equation (8) we can see that for different attribute orders, the candidate parents for $X_{i}$ may differ greatly. The joint distributions $\hat{P}\left(x,c\right)$ represented by KDBs learned from different attribute orders may not surely be same. The key issue for structure learning of restricted BNC is how to describe the most significant conditional dependence relationships among predictive attributes, or more precisely, the relationships between $X_{i}$ and its parent attribute $X_{j}$ (i>j). However for KDB, the attributes are sorted in descending order of $I(X_{i};C)$, which only considers the dependence relationship between $X_{i}$ and class variable *C* while neglecting the conditional dependence relationships between $X_{i}$ and its parents. If the first few attributes in the order are relatively independent of each other, the robustness of the network structure will be damaged from the beginning of structure learning. To address this issue, UKDB selects the parents of variable *C*, or $X_{p}$, which are also the parents of the other attributes from the viewpoint of Markov blanket. In addition, there exist strong conditional dependence relationships between $X_{p}$ and the other attributes. On the other hand, *k* corresponds to the maximum allowable degree of attribute dependence, thus the number of attributes in $X_{p}$ is *k*.

Suppose that attribute set $X_{p}$ contains *k* attributes $\left\{X_{n-k+1},\cdots ,X_{n}\right\}$ and the order of attributes in *X* is $\left\{X_{p},X_{1},\cdots ,X_{n-k}\right\}$, Formula (7) can be rewritten in another form,

(10) $$P\left(x,c\right)=P\left(x_{p}\right)P\left(c|x_{p}\right)\cdots P\left(x_{n-k}|x_{p},x_{1},\cdots ,x_{n-k-1},c\right)\;\left(k\geq 1\right)$$

The relationships between $X_{i}$ and its parents corresponding to Equations (7) and (10) are shown in Table 2.

Since $P(x_{p})$ is irrelevant to the classification, then

(11) $$P\left(c,x\right)\propto P\left(c|x_{p}\right)P\left(x_{1}|x_{p},c\right)\cdots P\left(x_{n-k}|x_{p},x_{1},\cdots ,x_{n-k-1},c\right)$$

**Figure 6:**
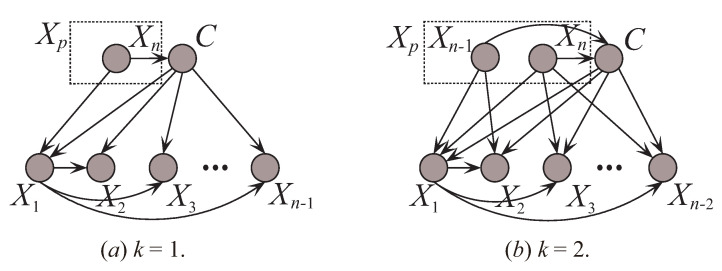
Two examples of UKDB when k=1 and k=2.

Thus, UKDB uses the following formula for classification,

(12) $$\begin{array}{c} c^{*}=\arg\max\overset{ˇ}{P}\left(x,c\right)=\arg\max P\left(c|x_{p}\right)\prod_{i=1}^{n-k}P\left(x_{i}|{\overset{ˇ}{\Pi }}_{i},c\right), \end{array}$$

where ${\overset{ˇ}{\Pi }}_{i}$ is one subset of $\left\{X_{p},X_{1},\cdots ,X_{i-1}\right\}$ and contains *k* attributes. For any attribute $X_{i}$ ($X_{i}\in X_{p}$), $X_{i}$ is the parent of the other attributes, then there should exist strong conditional dependencies, or tight coupling, between them. To this end, we sort the attributes by comparing the sum of CMI. To express this clearly in the following discussion, we sort the attributes by comparing the sum of CMI (SCMI) and $\mathrm{SCMI}\left(X_{i}\right)=\sum_{j}I\left(X_{i};X_{j}|C\right)\left(X_{i}\neq X_{j}\right)$. The first *k* attributes in the order with the largest SCMI are selected as $X_{p}$. To control the structure complexity, UKDB also require that $X_{i}$ should select at most *k* parents from $\Pi_{i}$ as shown in Table 2. The attribute sets $X_{c}$ and $X_{cp}$ will be determined thereafter. Figure 6 shows two examples of UKDB when k=1 and k=2.

In the real world, when attributes take different values the same dependence relationships between them may lead to wrong diagnosis or therapy. Considering attributes *Sex* and *Pregnant*, *Sex* = “Female” and *Pregnant* = “Yes” are highly related, whereas *Sex* = “female” and *Pregnant* = “No” also hold for some instances. Obviously, treatment of breast cancer during pregnancy should be different to that during non-pregnancy. CMI can weigh the conditional dependency between *Sex* and *Pregnant*, but cannot discriminately weigh the dependencies when these two attributes take different values. Target learning takes each testing instance $P=\{x_{1},\cdots ,x_{n},c=?\}$ as a target and tries to mine the dependence relationships between these attribute values [16]. From Equations (1) and (5), we have the following equations:

(13) $$\left\{\begin{array}{c} I\left(X_{i};C\right)=\sum_{x_{i}\in X_{i}}I\left(x_{i};C\right)\\ I\left(X_{i};X_{j}|C\right)=\sum_{x_{i}\in X_{i}}\sum_{x_{j}\in X_{j}}I\left(x_{i};x_{j}|C\right) \end{array}\right.$$

where

(14) $$\left\{\begin{array}{c} I\left(x_{i};C\right)=\sum_{c\in C}P\left(c,x_{i}\right)\log\frac{P(c,x_{i})}{P\left(c\right)P\left(x_{i}\right)}\\ I\left(x_{i};x_{j}|C\right)=\sum_{c\in C}P\left(x_{i},x_{j},c\right)\log\frac{P(x_{i},x_{j}|c)}{P\left(x_{i}|c\right)P\left(x_{j}|c\right)} \end{array}\right.$$

The definitions of MI and CMI are measures of the average dependence between attributes implicated in the training data. In contrast to those, local mutual information (LMI) $I(x_{i};C)$ and conditional local mutual information (CLMI) $I(x_{i};x_{j}|C)$ can weigh the direct dependence and conditional dependence relationships between attribute values implicated in each instance [16,24]. Similarly, we sort the attribute values by comparing the sum of CLMI (SCLMI) and $\mathrm{SCLMI}\left(x_{i}\right)=\sum_{j}I\left(x_{i};x_{j}|C\right)\left(x_{i}\neq x_{j}\right)$.

For Bayesian inference, LMI refers to the event when $X_{i}=x_{i}$ and can be used to measure the expected value of mutual dependence between $X_{i}$ and *C* after observing that $X_{i}=x_{i}$. CLMI can be used to weigh the conditional dependence between attribute values $x_{i}$ and $x_{j}$ while considering all possible values of variable *C*.

From Equations (1) and (5), to compute $I(X_{i};C)$ or $I(X_{i};X_{j}|C)$, all possible values of attribute $X_{i}$ need to be considered. If there exist missing or unknown value for attribute $X_{i}$ and $X_{j}$ in any instance, they will be replaced by some values and noise may be artificially introduced into the computation of $I(X_{i};C)$ or $I(X_{i};X_{j}|C)$. These missing or unknown values are regarded as noisy because the conditional dependence relationships between them and other non-noisy attribute values may be incorrectly measured. If the noisy part only account for a small portion of the non-noisy part, the dependence relationships learned from training data may be still of high-confidence level and the network structure of UKDB${}_{T}$ may be still robust. In contrast, from the definitions of LMI and CLMI (Equation (14)) we can see that for specific instance x, to compute $I(x_{i};C)$ or $I(x_{i};x_{j}|C)$ only these attribute values in x need to be considered. The computation of $I(x_{i};C)$ or $I(x_{i};x_{j}|C)$ concerning noisy values will not be needed. Thus, neglecting these noisy conditional dependence relationships may make the network structure of UKDB${}_{P}$ more robust.

We propose to use the Markov blanket and target learning to build an ensemble of two unrestricted BNCs, i.e., UKDB${}_{T}$ and UKDB${}_{P}$. UKDB${}_{T}$ and UKDB${}_{P}$ learn from different parts data space and their learning procedures are almost the same, thus they are complementary in nature. In the training phase, by calculating MI and CMI, UKDB${}_{T}$ describes the global conditional dependencies implicated in training data T. Correspondingly, in the classification phase, by calculating LMI and CLMI, UKDB${}_{P}$ describes the local conditional dependencies implicated in unlabeled testing instance P. Breiman [25] revealed that ensemble learning brings improvement in accuracy only to those “unstable” learning algorithms, in the sense that small variations in the training set would lead them to produce very different models. UKDB${}_{T}$ and UKDB${}_{P}$ are such algorithms. UKDB${}_{T}$ tries to learn the certain domain knowledge implicated in training dataset, whereas the domain knowledge may not describe the conditional dependencies in testing instance P. It may cause overfitting on the training set and underfitting on the testing instance. In contrast, UKDB${}_{P}$ can describe the conditional dependencies implicated in testing instance P, whereas the personalized knowledge is uncertain since the class label of P is unknown. It may cause underfitting on the training set and overfitting on the testing instance. Thus, an ensemble of UKDB${}_{T}$ and UKDB${}_{P}$ may be much more appropriate for making the final prediction.

The learning procedures of UKDB${}_{T}$ is described by Algorithm 1 as follows:

**Algorithm 1:** The UKDB${}_{T}$ algorithm

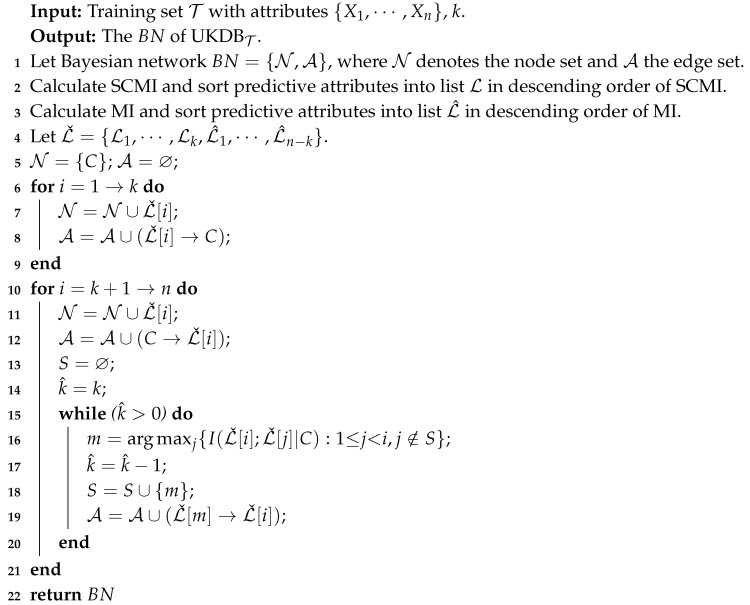



Since the class label of testing instance P is unknown, we can get all possible class labels from training set T. Assume that the probability the testing instance P in class *c* is 1/m for each $c\in \{c_{1},\cdots ,c_{m}\}$, there will be *m* “pseudo” instances. By adding these *m* “pseudo” instances to training set T, we can estimate the joint or conditional probabilities between arbitrary attribute value pairs by using Equation (14) to achieve the aim of learning conditional independence from a testing instance P.

The learning procedures of UKDB${}_{P}$ is shown in Algorithm 2, where “?” is represented the missing value in the dataset. To estimate the marginal and joint probabilities $P\left(c\right),P\left(x_{i},c\right)$ and $P(x_{i},x_{j},c)$, at training time UKDB needs one pass through the training data to collect the base statistics of co-occurrence counts. Calculating MI and CMI respectively need O(Nmnv) and $O(Nm{\left(nv\right)}^{2})$ time, where *N* is the number of training instances, *m* is the number of classes, *n* is the number of attributes and *v* is the number of values that discrete attributes may take on average. The procedure of parent assignment for each attribute needs $O(n^{2}logn)$. Thus, the time complexity for UKDB${}_{T}$ to build the actual network structure is $O(Nm{\left(nv\right)}^{2})$. Since UKDB${}_{P}$ only needs to consider the attribute values in the testing instance, calculating LMI and CLMI respectively need O(Nmn) and $O(Nmn^{2})$ time. The procedure of parent assignment for each attribute in UKDB${}_{P}$ needs the same time, $O(n^{2}logn)$. Thus, the time complexity for UKDB${}_{P}$ is only $O(Nmn^{2})$. UKDB${}_{T}$ and UKDB${}_{P}$ use different variations of P(x,c) to classify each single instance and corresponding time complexities are the same, O(mnk).

**Algorithm 2:** The UKDB${}_{P}$ algorithm

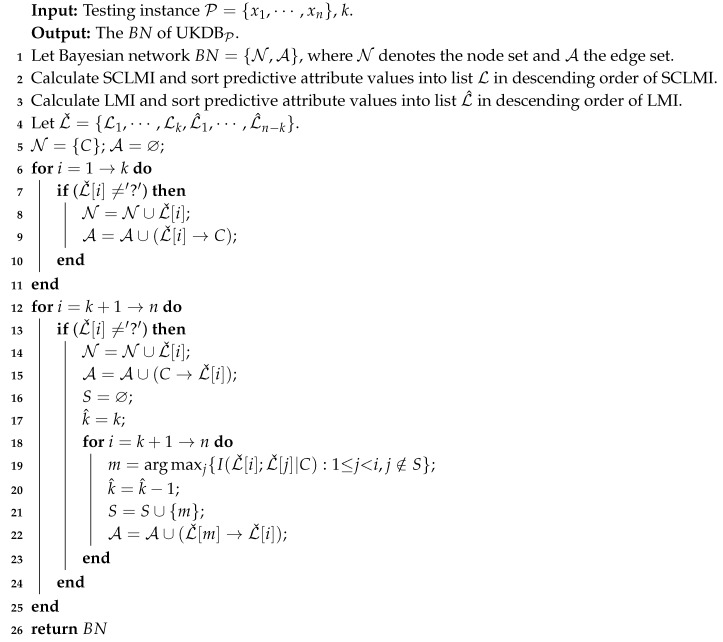



UKDB${}_{T}$ learned from training data T describes the general conditional dependencies, thus UKDB${}_{T}$ corresponds to the domain knowledge that may be suitable for most cases. In contrast, UKDB${}_{P}$ learned from testing instance P describes local conditional dependencies with uncertainty because all class labels are considered, thus UKDB${}_{P}$ corresponds to the personalized knowledge that may be suitable for P only [16].

When facing an expected case, it is difficult to judge which kind of knowledge should be considered in priority. Precision knowledge may provide some statistical information that the expert does not recognize and help him use the domain knowledge to confirm or rule out the decision. For different cases, the weights of UKDB${}_{P}$ and UKDB${}_{T}$ may differ greatly. In this paper, without any prior knowledge we simply use the uniformly weighted average instead of the nonuniformly weighted one. The final probability estimate for the ensemble of UKDB${}_{T}$ and UKDB${}_{P}$ is,

$$\hat{P}\left(c|x\right)=\frac{P\left(c|x,{\mathrm{UKDB}}_{T}\right)+P\left(c|x,{\mathrm{UKDB}}_{P}\right)}{2}.$$

## 4. Results and Discussion

### 4.1. Data

Breast cancer is the leading life-threatening cancer for women, especially for those aged between 40 and 55 in US and Europe [26]. American Cancer Society (ACS) estimated that [27], in 2017 about 252,000 women were diagnosed with invasive breast cancer and over 60,000 with noninvasive breast cancer. Sometimes it is too late for those women to be treated since no obvious symptoms appear before the diagnosis and among them about 12.8% will die of breast cancer after diagnosis [27]. Thus, there is strong demand for improved classification/detection systems in medical science community.

Dr William H. Wolberg collected data relevant to breast cancer during his stay at the University of Wisconsin-Madison Hospitals from 1989 to 1991, and provided the data to the UCI repository of machine learning [17]. This WBC database is relatively small, containing only 699 instances of breast cancer. In this database, 458 (65.5%) instances are benign and 241 (34.5%) instances are malignant. Each instance has 10 predictive attributes and the detailed introduction of the 10 attributes is shown in Table 3. Please note that some instances have missing values. In addition, attribute “Sample code number” is not considered in experimental study because it represents the id number and is not helpful for classification.

In the last decade, larger datasets are not scarce resources anymore [28,29,30]. Larger data quantities can help make the estimation of conditional probabilities more accurate. BNCs need higher-degree representation of attribute dependence and more accurate estimation of probability distribution to deal with them. Ten large datasets (size > 3000) with different number of attributes (n≥10) are selected from the UCI repository of machine learning [17] for experimental study. Table 4 describes the details of each dataset, including the number of instances, attributes and classes.

### 4.2. Evaluation Function

In machine learning, zero-one loss [31] is one of the standard measures for evaluating the classification performance. The bias-variance decomposition [32] for zero-one loss can help analyze the expected generalization error of trained models. To achieve bias-variance trade-off is a key issue in supervised learning. Zero-one loss can measure the extent to which a classifier correctly identifies the class label of an unlabeled instance. Given *M* testing instances, the zero-one loss function can be calculated as follows:

(15) $$\xi \left(c,\hat{c}\right)=\frac{\sum_{i=1}^{M}\left\{1-\delta \left(c_{i},\hat{c_{i}}\right)\right\}}{M},$$

where $c_{i}$ and $\hat{c_{i}}$ are respectively the true class label and predicted label of the *i*-th instance, besides $\delta (c_{i},\hat{c_{i}})=1$ if $c_{i}=\hat{c_{i}}$ and 0 otherwise. While dealing with highly imbalanced datasets where “positive” class has very low proportion as compared to the “negative” class, F1 score can help to judge whether the classifier tends to be biased towards the majority class or not. The F1 score is defined as follows,

(16) $$F1=\frac{2TP}{2TP+FP+FN}$$

where TP is equal to the number of positive instances that have been classified correctly, FP and FN are equal to the numbers of positive instances that have been misclassified and the numbers of negative instances that have been misclassified.

We also has been introduced the ROC (Receiver Operating Characteristics) cure [33,34] to evaluate performance of machine-learning algorithms. The ROC curve is created by plotting the true-positive rate (TPR) against the false-positive rate (FPR) at various threshold settings. The TPR is also known as sensitivity or recall in machine learning. The FPR is also known as the fall-out or probability of false alarm and can be calculated as (1 - specificity), where specificity is the true negative rate (TNR). All formula involved are defined as follows:

(17) $$TPR=\frac{TP}{TP+FN}$$

(18) $$TNR=\frac{TN}{TN+FP}$$

(19) $$FPR=\frac{FP}{FP+TN}=1-TNR$$

We compared the proposed algorithm when k=1,2 with several benchmark classifiers [12,13,23] that were presented in the literature. The statistical results of all evaluated functions using 20 rounds of 10-fold cross validation are shown in Table 5. For each fold, 9/10 of the data was used for training and 1/10 of the data was used for testing. In addition, all experiments have been conducted on a desktop computer with an Intel(R) Xeon(R) CPU X5680 @ 3.33GHz, 64 bits and 8192 MiB of memory. In addition, for training data, missing values for qualitative attributes are replaced with modes and those for quantitative attributes are replaced with means from the training data [35,36,37]. In addition, for testing data, UKDB${}_{P}$ proposes a natural way for dealing with missing values, not considering the dependence relationships related to missing values. The negative effect caused by missing values for UKDB${}_{P}$ can be mitigated by removing noisy dependence relationships, and the learned network structure may be more robust.

Sampling is one of the main methods used for handling the problem of imbalanced dataset, which follows two different approaches: undersampling and oversampling [38,39,40]. Undersampling methods aim to decrease the size of the majority class. On the contrary to undersampling, oversampling algorithms tend to balance class distributions through the increase of the minority class. Since undersampling may cause the classifier to miss important concepts pertaining to the majority class, we conduct all experiments with oversampling. In the preprocessing stages of datasets, we add a set of randomly selected minority instances in the set of minority class instances and augment the original set by replicating the selected instances and adding them to it. In this way, the number of total instances in the set of minority class instances is increased and the class distribution balance is adjusted accordingly.

We also employ the Win/Draw/Loss records to summary the experimental results. Cell[i,j] in each table contains the number of datasets for which the BNC on the *i*th-row performs better (Win), equally well (Draw) or worse (Loss) than the other on the *j*th-column. In the following experiments, we assess a difference as significant if the outcome of a one-tailed binomial sign test is less than 0.05.

### 4.3. Experimental Study on WBC Dataset

From Table 5 we can see that except NB, UKDB (k=1) has a remarkably obvious prediction superiority compared to the other algorithms in terms of zero-one loss and UKDB (k=2) achieves slightly improved F1 score than other algorithms. Although NB achieves lower errors than other algorithms on WBC, it is just a special case. As Sahami [13] argued that there would be expected to achieve optimal Bayesian accuracy if more “right” dependencies are captured. In most cases, BNCs with simple structure perform worse than those with complex structure. We will further demonstrate it in the Section 4.4.3.

UKDB${}_{T}$, which is learned from all training instances, can describe the general conditional dependence relationships. However, it is not all the dependence relationships but only some of them that may hold for a certain instance. In contrast, UKDB${}_{P}$ can encode the most possible local conditional dependencies implicated in one single testing instance. UKDB can use the knowledge learned from the training set and testing instances by applying the aggregating mechanism. If UKDB${}_{T}$ and UKDB${}_{P}$ are complementary to each other for classification, an ideal phenomenon is that they focus on different key points. To prove this, we take an instance from WBC dataset for case study, and the detail of the instance is shown as follows,

$$P=\{x_{1}=9,x_{2}=5,x_{3}=8,x_{4}=1,x_{5}=2,x_{6}=3,x_{7}=2,x_{8}=1,x_{9}=5\}$$

By comparing MI $I(X_{i};C)$, $\bar{X}=\left\{X_{2},X_{3},X_{6}\right\}$ are the first three key attributes for UKDB${}_{T}$. Whereas by comparing $I(x_{i};C)$, $\hat{X}=\{X_{4},X_{5},$ $X_{8}\}$ are the first three for UKDB${}_{P}$. The marginal probabilities of each attribute value in P are shown in Table 6. From Table 6, for any attribute value $x_{i}$ ($X_{i}\in \hat{X}$) and $x_{j}$ ($X_{j}\in \bar{X}$), $P\left(x_{i}\right)>P\left(x_{j}\right)$ always holds. Then for attribute $X_{k}$, it is more possible that $P\left(x_{k}|x_{i},c\right)>P\left(x_{k}|x_{j},c\right)(k\neq i$ and k≠j). To maximize the joint probability P(x,c), as (10) suggests, an ideal condition is that each underlying conditional probability will be maximized. Obviously, UKDB${}_{P}$ can achieve a much more reasonable attribute order.

**Figure 7:**
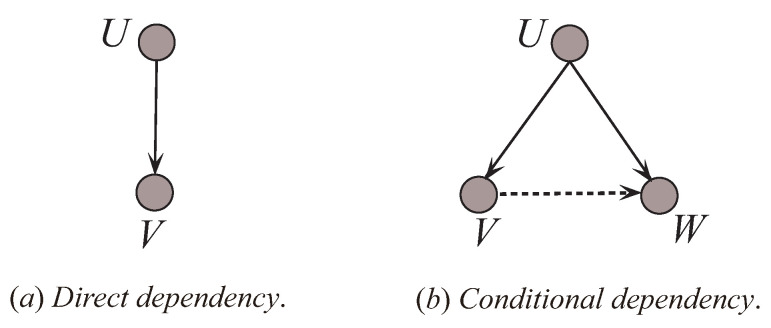
The dependency types in BNCs.

Generally, as Figure 7 shows, dependency types in BNCs can be divided into two types: one is the direct dependence relationship (indicated in the Figure 7a by the solid line), such as the relationships between variables *U* and *V*; another is the conditional dependence relationship (indicated in the Figure 7b by the dotted line), such as the relationships between variables *V* and *W* given *U*. To interpret the effect of dependency types to UKDB, a simulation study has been carried out on dataset WBC.

**Figure 8:**
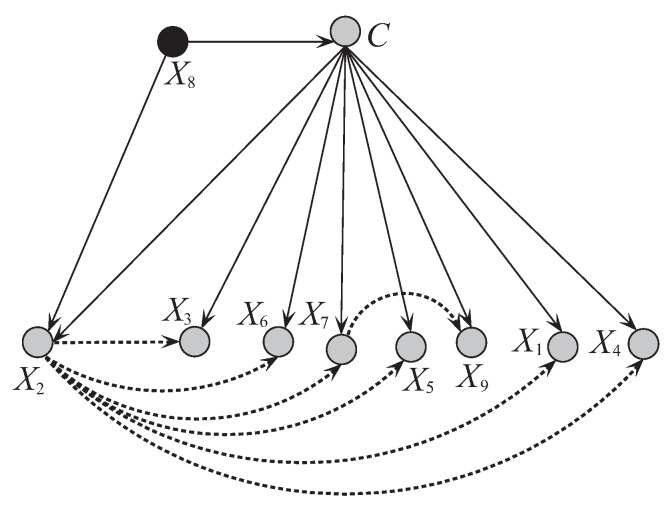
The network structure of UKDB${}_{T}$ corresponding to breast cancer dataset.

**Figure 9:**
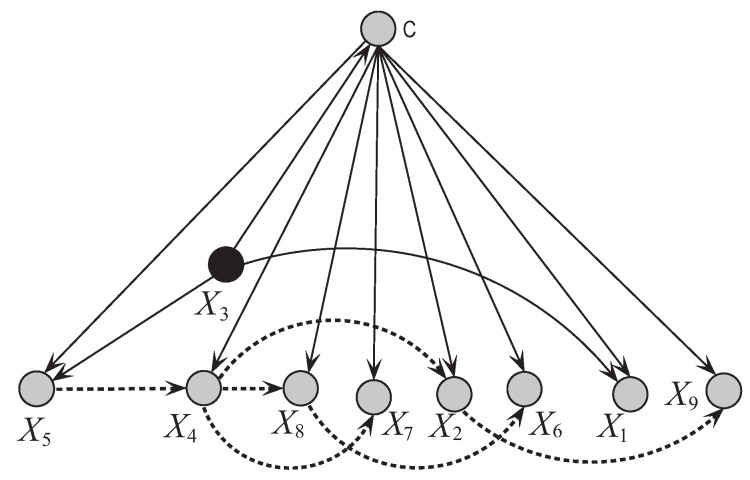
The network structure of UKDB${}_{P}$ corresponding to testing instance P = {9,5,8,1,2,3,2,1,5} in breast cancer dataset.

**Figure 10:**
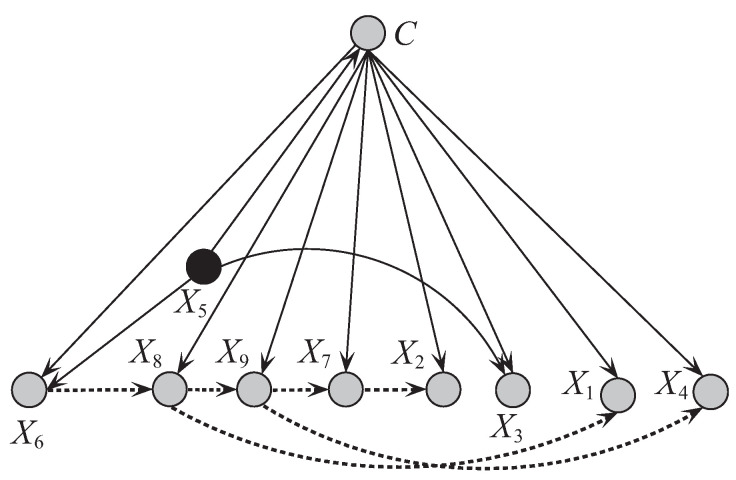
The network structure of UKDB${}_{P}$ corresponding to testing instance P’ = {5,3,3,3,6,10,3,1,1} in breast cancer dataset.

Figure 8 and Figure 9 respectively show the network structures of UKDB${}_{T}$ and UKDB${}_{P}$ on dataset WBC when k=1, where UKDB${}_{P}$ is based on testing instance P. The parent attribute of class variable is annotated in black. We can see clearly the differences in direct and conditional dependencies between them. For UKDB${}_{T}$, attribute $X_{8}$ and class *C* have direct dependence relationships with other attributes, and $X_{2}$ is the key attribute that has conditional dependence relationships with almost all the other attributes. In contrast, for UKDB${}_{P}$, $X_{3}$ and *C* have direct dependence relationships with other attributes, and $X_{4}$ plays the main role instead and is the common parent of only 3 out of 8 other attributes. In Figure 10 another structure is presented for the testing instance $P^{\prime }=\left\{5,3,3,3,6,10,3,1,1\right\}$ that is different from the structure obtained for instance P={9,5,8,1,2,3,2,1,5}. These examples illustrate the personalized structure (e.g., Figure 9) generated from our targeted learning for given testing instance are discriminative not only with the domain structure (e.g., Figure 8) but also other personalized structure (e.g., Figure 10) learned from other testing instance. In the next section, we will prove that the ensemble of these discriminative BNCs can use the knowledge learned from the training set and testing instances to achieve better classification performance.

### 4.4. Further Experiments on Other Datasets

#### 4.4.1. The Effect of Values of *k*

We firstly compared the classification performance of KDB and UKDB with the same values of *k*. Since the restrictions of currently available hardware place some requirements on the software and the complexity of the probability table increases exponentially as *k* increases, to achieve the trade-off between classification performance and efficiency, we respectively compared KDB and UKDB with k=1 and k=2 on 10 datasets (described in Table 4). The detailed results in terms of zero-one loss can be found in Table A1 in Appendix A.

As shown in Table 7, for UKDB, the model with k=2 achieves significant advantages over the one with k=1 and results in Win/Draw/Loss of 6/2/2. In addition, there are only two datasets, i.e., Dis and Mushroom, have larger results of zero-one loss with UKDB, which indicates that UKDB (k=2) seldom performs worse than UKDB (k=1). In addition, for many datasets, UKDB (k=2) substantially improved the classification performance of UKDB (k=1), for example, the decrease from 0.0644 to 0.0414 for the datasets Adult.

#### 4.4.2. The Effect of Missing Values

As mentioned above, for training data, missing values for qualitative attributes are replaced with modes and those for quantitative attributes are replaced with means from the training data [35,36,37]. In addition, for testing data, UKDB${}_{P}$ proposes a natural way for dealing with missing values, not considering the dependence relationships related to missing values. The negative effect caused by missing values for UKDB${}_{P}$ can be mitigated by removing noisy dependence relationships, and the learned network structure may be more robust.

In this section, to prove that UKDB has the ability to mitigate the negative effect caused by missing values in testing instance, we also present a simulation experiment to investigate the effect of missing values to UDKB. We choose datasets with no missing values from Table 4. In addition, there are three datasets satisfying this conditions, i.e., Chess, Magic and Spambase. To compare the algorithm on a controlled situation, when classifying testing instances, we manually and randomly delete 5% of attribute values in each instance.

Table 8 shows the detailed results of UKDB (k=2) on two sets of data with and without missing values in terms of zero-one loss. As can be seen, although some attribute values of testing instances have been deleted, the results of zero-one loss on these 3 datasets are similar to the one without missing values (we assess a difference as significant if the outcome of a one-tailed binomial sign test is less than 0.05), i.e., UKDB has the ability to mitigate the negative effect caused by missing values in testing instance.

#### 4.4.3. The Effect of Criterion Used to Measure the Strength of the Dependence between the Variables

Our proposed algorithm, UKDB, is using MI and CMI (or LMI and CLMI) to measure the strength of the dependence between attributes. Actually, UKDB could use others. Since the efficiency of the UKDB depends on the efficiency of MI and CMI, we use another criterion, pointwise mutual information (PMI) and pointwise conditional mutual information (PCMI) to compare and to show in which situations MI and CMI is more (or less) efficient. In contrast to MI and CMI, PMI and PCMI refer to single events, whereas MI and CMI refer to the average of all possible events [41]. In computational linguistics, PMI and PCMI have been used for finding collocations and associations between words [41]. They can be calculated as follows:

(20) $$\mathrm{PMI}\left(x;c\right)=log\frac{P(x,c)}{P(x)P(c)}.$$

(21) $$\mathrm{PCMI}\left(x_{i};x_{j}|c\right)=log\frac{P(x_{i},x_{j}|c)}{P\left(x_{i}|c\right)P\left(x_{j}|c\right)}.$$

Table 9 shows the Win/Draw/Loss comparison results of UKDB (k=2) with {MI, CMI} and {PMI, PCMI}. The corresponding detailed results can be found in Table A2 in Appendix A. As can be seen, UKDB (k=2) with {MI, CMI} achieves lower error more often than the one with {PMI, PCMI}. To identify the efficiency between UKDB (k=2) with different information-based criteria to measure the dependence relationships between attributes, we present the results of average running computational time for UKDB (k=2) with {MI, CMI} and {PMI, PCMI} in Table 10. The results in Table 10 reinforce what the orders of complexity for these two algorithms indicated, i.e., UKDB (k=2) with {MI, CMI} needs more time to build model than the one with {PMI, PCMI} on most datasets. For example, on dataset Census-Income, the running computational time of UKDB with {PMI, PCMI} is almost 1.84 times faster than the one with {MI, CMI} (as highlighted in bold in the table). Thus, although UKDB with {PMI, PCMI} is more efficient than the one with {MI, CMI} in terms of average running computational time, UKDB with {MI, CMI} has better classification performance in terms of zero-one loss at the cost of increasing less computational time.

#### 4.4.4. UKDB vs. NB, TAN and KDB

Although NB ranked the highest among all algorithms on WBC database in terms of zero-one loss and F1, the conditional independence assumption of NB is not true in most cases, furthermore, many researchers found that general algorithm performs better than NB in most cases [12,13,18,19,20]. Thus, it is necessary to have more general algorithm even if NB works the best in some cases.

In this section, we will demonstrate that the advantages of UKDB are due to its flexible high-dependence representation when dealing with large datasets. Since UKDB with k=2 achieves lower results of zero-one loss more often than the one with k=1, we compare UKDB (k=2) with other lower-dependence BNCs, i.e., NB (0-dependence) and TAN (1-dependence). The experimental results of KDB (2-dependence when k=2) are also shown for object reference. The derailed results of the average zero-one loss, bias and variance on 10 datasets (described in Table 4) are presented in Appendix A, respectively.

Table 11 shows the corresponding Win/Draw/Loss comparison results of different BNCs.

The results of zero-one loss in Table 11 reveal some patterns that confirm the hypothesis proposed above. As can be seen, TAN performs better than NB on 8 datasets and never worse. KDB performs better than TAN on 5 datasets and never worse. UKDB performs the best among all classifiers. It proved that the superior classification performance of NB on dataset WBC is just a special case. NB, TAN, KDB and UKDB can represent different degrees of dependence relationship. In general, as structure complexity increases, higher-dependence BNCs enjoy significant advantage in classification over lower-dependence BNCs on most cases.

From Table 11, in terms of bias, TAN still performs better than NB, and KDB performs better than TAN. However, the advantage of UKDB over KDB is not so significant. Higher-dependence BNCs can represent more conditional dependencies, which in general help these models to approximate the correct value of conditional probability $P(x_{i}|\Pi_{i},c)$. From Table 11, in terms of variance, NB achieves the lowest variance because there exists no structure learning for it and its structure remains the same regardless of the change of training data. TAN performs better than KDB on 5 datasets and worse on 3 datasets. UKDB performs better than TAN on 5 datasets and worse on 3 datasets, and it performs better than KDB on 7 datasets and worse on 2 datasets. This also emphasizes that the robustness of UKDB is only second to NB. UKDB enjoys significant advantage over TAN and KDB in terms of bias and variance. Simple network structure may result in underfitting whereas complex one may result in overfitting. It is very difficult for a BNC to achieve the trade-off between structure complexity and classification performance. However, mining the possible dependence relationships implicated in testing instance helps to alleviate the negative effect caused by overfitting while improving the classification accuracy.

To attest the effective superiority of the UKDB, we use the Friedman test [42] for comparison of all alternative algorithms on other 10 datasets in Table 4. The null hypothesis of the Friedman test is that there is no difference in average ranks. With 4 algorithms and 10 datasets, the Friedman test is distributed according to the *F* distribution with 4−1=3 and (4−1)×(10−1)=27 degrees of freedom. The critical value of F(3,27) for α=0.05 is 2.9603. The result of Friedman test for zero-one loss is 22.25>2.9603 with p<0.001. Hence, we reject the null hypothesis. That is to say, the seven algorithms are not equivalent in terms of zero-one loss results. The average ranks of zero-one loss of different classifiers are {NB(3.8000), TAN(2.8000), KDB(2.2000), UKDB(1.2000)}, and the minimum required difference of mean rank is 0.6701, i.e., the rank of UKDB is better than that of other algorithms, followed by KDB, TAN and NB. UKDB has significant statistical difference with NB, TAN and KDB.

**Figure 11:**
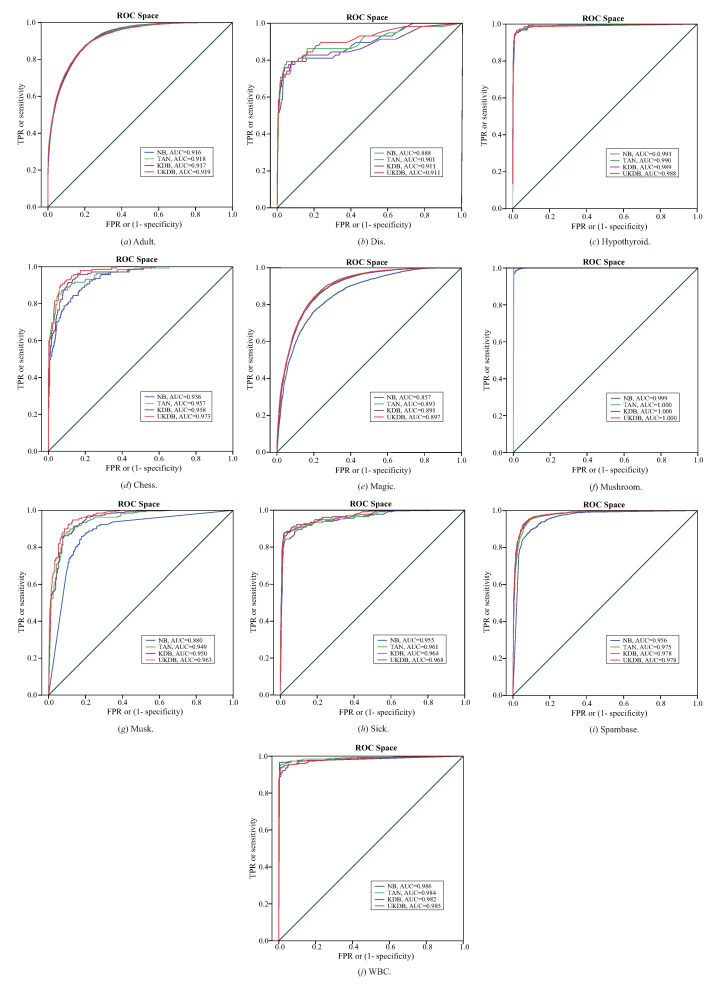
The ROC cures for NB, TAN, KDB (k=2) and UKDB (k=2) on 10 datasets.

The ROC cures for NB, TAN, KDB (k=2) and UKDB (k=2) on 10 datasets are presented in Figure 11, respectively. The X-axis represents (1 - specificity) and Y-axis represents sensitivity. The area under the curve (AUC) is an effective and combined measure of sensitivity and specificity for assessing inherent validity of a diagnostic test [33]. The value of AUC closer to 1 indicates better performance of the test. According to the values of AUC, UKDB performs lower results more often than other algorithms, especially on datasets Adult, Chess, Magic, Musk and Sick. Compared with KDB, UKDB achieves similar values of AUC on 4 datasets (Dis, Hypothyroid, Mushroom and Spambase), i.e., UKDB also has significant advantages with NB, TAN and KDB in terms of ROC cures.

To further demonstrate the performance of UKDB over KDB, we employ the goal difference (GD) [19,21]. Suppose there are two classifiers *A* and *B*, the value of GD can be computed as follow:

(22) GD(A;B|T)=|win|−|loss|,

where T is the datasets, |win| and |loss| represent the number of datasets on which *A* performs better or worse than *B*, respectively.

**Figure 12:**
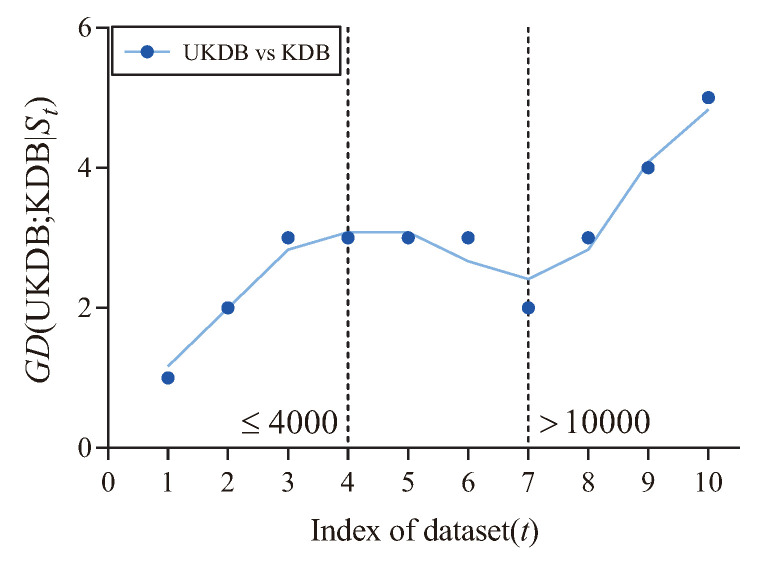
The fitting curve of GD (UKDB;KDB|$S_{t}$) in terms of 0-1 loss.

Figure 12 shows the fitting curve of GD (UKDB;KDB|$S_{t}$) in terms of 0-1 loss. The X-axis shows the indexes of different datasets, referred to as *t*, which correspond to that described in Table 4. In addition, the Y-axis corresponds to the value of GD (UKDB;KDB|$S_{t}$), where $S_{t}=\left\{D_{m}|m\leq t\right\}$ and $D_{m}$ is the dataset with index *m*. As can be seen, UKDB enjoys significant advantages over KDB in terms of 0-1 loss when the number of instances ≤4000 (3 wins and 1 draw) or >10,000 (3 wins), otherwise the advantage is not significant (2 draws and 1 loss).

**Figure 13:**
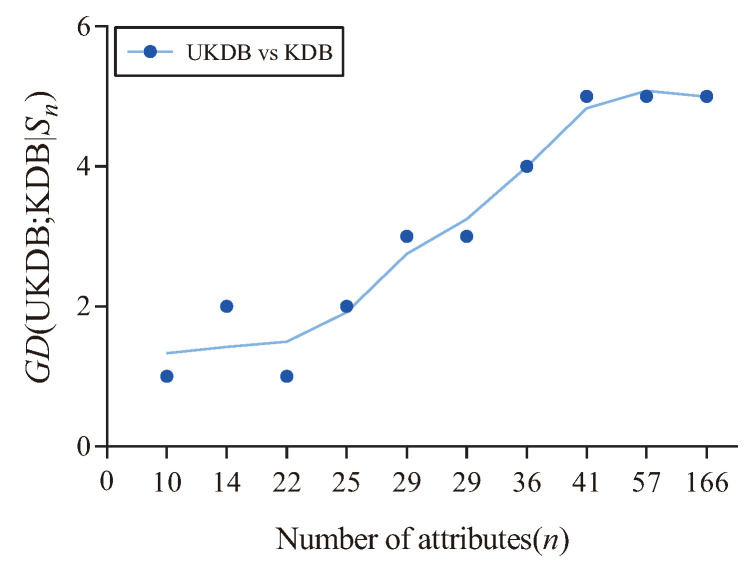
The fitting curve of GD (UKDB;KDB|$S_{n}$) in terms of 0-1 loss.

Figure 13 shows the fitting curve of GD (UKDB;KDB|$S_{n}$) in terms of 0-1 loss. The X-axis shows the number of attributes for different datasets, referred to as *n*, which correspond to that described in Table 4. In addition, the Y-axis corresponds to the value of GD (UKDB;KDB|$S_{n}$), where $S_{n}=\left\{D_{n^{\prime }}|n^{\prime }\leq n\right\}$ and $D_{n^{\prime }}$ is the dataset with $n^{\prime }$ attributes. We can see that when the number of attributes >22, the advantage of UKDB over KDB is significant in terms of 0-1 loss (4 wins and 3 draws), otherwise the advantage is not significant (2 wins and 1 loss).

#### 4.4.5. UKDB vs. Target Learning

Target learning [16] is a framework that takes each unlabeled testing instance P as a target and builds a specific Bayesian model BNC${}_{P}$ to complement BNC${}_{T}$ learned from training data T. It respectively uses TAN and KDB as the base classifier to clarify the superiority of target learning (which referred to as TAN^e^ and KDB^e^).

We have conducted experiments with TAN^e^ and KDB^e^ (k=2) on 10 datasets (described in Table 4). The detailed zero-one loss results of all alternative algorithms are presented in Table A6 in Appendix A. Table 12 shows the Win/Draw/Loss comparison results of TAN^e^, KDB^e^ and UKDB (k=2) in terms of zero-one loss. As can be seen, UKDB achieves lower values of zero-one loss more often than TAN^e^ and KDB^e^, for example, the decrease from 0.4821 ± 0.0037 (TAN^e^) or 0.4781 ± 0.0039 (KDB^e^) to 0.1537 ± 0.0045 (UKDB) for the dataset Abalone.

The Friedman test was also performed for these three algorithms on 10 datasets. The final result is 5.6862 >F(2,18)=3.5546 with p<0.001. This means that at α=0.05, there is evidence to reject the null hypothesis that all algorithms are equivalent. The average ranks of zero-one loss of these three algorithms are {TAN${}^{e}\left(2.4500\right)$, KDB${}^{e}\left(2.2500\right)$, UKDB(1.3000)}, and the minimum required difference of mean rank is 0.7655, which demonstrates that UKDB has significant statistical difference with TAN^e^ and KDB^e^.

#### 4.4.6. UKDB vs. ETAN

Cassio P. de Campos et al. [43] proposed an extended version of the TAN, ETAN, which also does not require attributes to be connected to the class. Based on a modification of Edmonds’ algorithm, its structure learning procedure explores a superset of the structures that are considered by TAN, yet achieves global optimality of the learning score function in a very efficient way.

Since it shares similarities with UKDB (k=1), we have conducted experiments with ETAN on 10 datasets (described in Table 4). The detailed zero-one loss results can be found in Table A7 in Appendix A. The Win/Draw/Loss comparison results are presented in Table 13. As can be seen, UKDB obtains lower error than ETAN more often than the reverse. Although ETAN is an efficient algorithm and has similar unrestricted Bayesian network structure with UKDB (k=1), it is a single model. On the contrary, UKDB is an ensemble algorithm.

The corresponding results of Friedman test for these three algorithms on 10 datasets is 4.0435>F(2,18)=3.5546 with p<0.001. The corresponding average ranks in terms of zero-one loss are {ETAN(2.5000), UKDB (k=1)(2.1000), UKDB(k=2)(1.3000)}, and the minimum required difference of mean rank is 0.8227, which demonstrates that the rank of UKDB (k=2) is better than that of other algorithms, followed by UKDB (k=1) and ETAN. UKDB (k=2) has significant statistical difference with ETAN.

## 5. Conclusions

In this paper, we have proposed to extend KDB from restricted BNC to unrestricted one by applying Markov blanket. The final classifier, called UKDB, demonstrates better classification performance with high expressivity, enhanced robustness and tight coupling. For each testing instance P, an appropriate local Bayesian classifier UKDB${}_{P}$ is built using the same learning strategy as that of UKDB${}_{T}$ learned from training data T. Compared with other state-of-the-art BNCs, the novelty of UKDB is that it can use the information mined from labeled and unlabeled data to make joint decisions. From the case study we can see that given testing instances $P_{1}$ and $P_{2}$, the weights of dependence relationships between the same pair of attribute values may differ that makes the topology of UKDB${}_{P_{1}}$ distinguish from that of UKDB${}_{P_{2}}$. Besides, the model is learned directly from the data in some field, and it can only express part of domain knowledge, i.e., datasets are only part of the field, and the knowledge of statistics may be contrary to expert knowledge. Some of the mined knowledge does not conform to the knowledge of medical experts, which requires the discrimination of expert knowledge. Thus, if given expertise in medicine, the network structures of UKDB${}_{P}$ and UKDB${}_{T}$ will be improved.

Given a limited number of instances, the accuracy of probability estimation determines the robustness of dependence relationships, and then determines the structure complexity of BNCs. The characteristic of tight coupling helps UKDB improve the probability estimation. UKDB has been compared experimentally with some state-of-the-art BNCs with different structure complexities. Although KDB and UKDB are of the same structure complexity, UKDB presents superior advantage over KDB in terms of classification accuracy (zero-one loss) and robustness (bias and variance). The independence assumption of NB rarely holds for all instances but may hold for specific instance. However, high-dependence BNCs, e.g., TAN, KDB and UKDB focus on the interdependence between attributes but disregard the independence between attribute values. If the independence in testing instance can be measured and identified, UKDB${}_{P}$ can provide a much more competitive representation.

Target learning is related to dependence evaluation when attributes take specific values. Because the proposed UKDB${}_{P}$ is based on UKDB, it needs enough data to learn accurate conditional probability during structure learning. Thus, in practical applications, the inaccurate estimate of conditional probability for some attribute values, e.g., $P(x_{i}|\Pi ,c)$, may lead to noise propagation in the estimate of joint probability P(c,x). This situation is more obvious while dealing with datasets with less attributes. Therefore, our further research is to decide the appropriate estimate of conditional probability needed for this purpose and to seek alternative methods, e.g., Laplace correction.

## Figures and Tables

**Table 1:** List of symbols used.

Notation	Description
P(·)	probability estimation
$X_{i}$	predictive attribute (or variable)
$x_{i}$	discrete values for attribute $X_{i}$
$x=(x_{1},\cdots ,x_{n})$	an instance of *n*-dimensional vector
*C*	class variable
*c*	discrete values for *C*
$\Omega_{C}$	set of labels of the class variable *C*
*N*	number of training instances
*M*	number of testing instances
*n*	number of predictive attributes
$D=(<x^{1},c^{1}>\cdots ,<x^{N},c^{N}>)$	training dataset
$<x^{i},c^{i}>$	the *i*-th training instance with the corresponding class label

**Table 2:** The relationships between $X_{i}$ and its parents corresponding to the restricted and unrestricted BNC.

Relationships in the Restricted BNC		Relationships in the Unrestricted BNC	
$X_{i}$	$\Pi_{i}$	$X_{i}$	$\Pi_{i}$
*C*	{}	*C*	$\left\{X_{p}\right\}$
$X_{1}$	{C}	$X_{1}$	$\left\{X_{p},C\right\}$
$X_{2}$	$\left\{X_{1},C\right\}$	$X_{2}$	$\left\{X_{p},X_{1},C\right\}$
$X_{3}$	$\left\{X_{1},X_{2},C\right\}$	$X_{3}$	$\left\{X_{p},X_{1},X_{2},C\right\}$
⋮	⋮	⋮	⋮
$X_{n}$	$\left\{X_{1},X_{2},\cdots ,X_{n-1},C\right\}$	$X_{n-k}$	$\left\{X_{p},X_{1},\cdots ,X_{n-k-1},C\right\}$

**Table 3:** Attributes in WBC database.

Attribute	Type	Explanation	Symbol
Sample code number	Discrete	code number	− −
Clump Thickness	Discrete	[1,10]	$X_{1}$
Cell Size	Discrete	[1,10]	$X_{2}$
Cell Shape	Discrete	[1,10]	$X_{3}$
Marginal Adhesion	Discrete	[1,10]	$X_{4}$
Epithelial Cell Size	Discrete	[1,10]	$X_{5}$
Bare Nuclei	Discrete	[1,10]	$X_{6}$
Bland Chromatin	Discrete	[1,10]	$X_{7}$
Normal Nucleoli	Discrete	[1,10]	$X_{8}$
Mitoses	Discrete	[1,10]	$X_{9}$
Class	Binary	2 for benign,	*C*
		4 for malignant	

**Table 4:** Datasets.

No.	Dataset	Instance	Attribute	Class
1	Hypothyroid	3163	25	2
2	Chess	3196	36	2
3	Dis	3772	29	2
4	Sick	3772	29	2
5	Spambase	4601	57	2
6	Musk	6598	166	2
7	Mushroom	8124	22	2
8	Magic	19,020	10	2
9	Adult	48,842	14	2
10	Census-Income	299,285	41	2

**Table 5:** Comparison of various algorithms from literature based on the WBC dataset.

Algorithms	Reference	Zero-One Loss	*F*1 Score
NB	Duda and Hart et al. (1973) [11]	0.0258	0.8006
TAN	Friedman et al. (1997) [12]	0.0429	0.7858
KDB (*k* = 1)	Sahami (1996) [13]	0.0485	0.7865
KDB (*k* = 2)	Sahami (1996) [13]	0.0521	0.7869
UKDB (*k* = 1)		0.0301	0.7917
UKDB (*k* = 2)		0.0385	0.7932

**Table 6:** Attribute values in P and corresponding marginal probabilities.

*x_i_*	*x*_1_ = 9	*x*_2_ = 5	*x*_3_ = 8	*x*_4_ = 1	*x*_5_ = 2	*x*_6_ = 3	*x*_7_ = 2	*x*_8_ = 1	*x*_9_ = 5
$P(x_{i})$	0.0200	0.0429	0.0401	0.5823	0.5522	0.0401	0.2375	0.6338	0.0086

**Table 7:** Win/Draw/Loss comparison results of UKDB (*k* = 1) and UKDB (*k* = 2) in terms of zero-one loss.

Win/Draw/Loss	UKDB (*k* = 1)
UKDB (*k* = 2)	6/2/2

**Table 8:** Detailed results of UKDB (*k* = 2) on two sets of data with and without missing values in terms of zero-one loss.

	Results with Missing Values	Results without Missing Values
Chess	0.04247 ± 0.0071	0.0414 ± 0.0061
Magic	0.2001 ± 0.0203	0.1987 ± 0.0101
Spambase	0.0760 ± 0.0153	0.0732 ± 0.0144

**Table 9:** Win/Draw/Loss comparison results of UKDB (*k* = 2) with {MI, CMI} and {PMI, PCMI}.

Win/Draw/Loss	UKDB (*k* = 2) with {PMI, PCMI}
UKDB (*k* = 2) with {MI, CMI}	5/5/0

**Table 10:** The average results of running computational time for UKDB (*k* = 2) with {MI, CMI} and {PMI, PCMI}.

Datasets	Time (s)	
	UKDB (*k* = 2) with {MI, CMI}	UKDB (*k* = 2) with {PMI, PCMI}
Hypothyroid	0.1139	0.0688
Chess	0.0641	0.0368
Dis	0.1969	0.1172
Sick	0.1999	0.1203
Spambase	2.0921	1.1009
Musk	9.4360	4.7562
Mushroom	0.2656	0.1631
Magic	0.1420	0.1117
Adult	0.7436	0.5131
**Census-Income**	**83.6734**	**45.5719**
Total	5.2560	9.6928

**Table 11:** The Win/Draw/Loss comparison results of different BNCs in terms of zero-one loss, Bias and Variance.

	Classifier	NB	TAN	KDB (*k* = 2)
	TAN	8-2-0		
0-1 loss	KDB (*k* = 2)	9-1-0	5-5-0	
	UKDB (*k* = 2)	10-0-0	7-2-1	6-3-1
	TAN	9-0-1		
Bias	KDB (*k* = 2)	9-0-1	5-5-0	
	UKDB (*k* = 2)	9-0-1	6-4-0	2-8-0
	TAN	3-0-7		
Variance	KDB (*k* = 2)	4-0-6	3-2-5	
	UKDB (*k* = 2)	3-2-5	5-3-2	7-1-2

**Table 12:** Win/Draw/Loss comparison results of TAN^*e*^, KDB^*e*^ and UKDB (*k* = 2) in terms of zero-one loss.

Win/Draw/Loss	TAN^*e*^	KDB^*e*^ (*k* = 2)
UKDB (*k* = 2)	7/2/1	6/4/0

**Table 13:** Win/Draw/Loss comparison results of ETAN, UKDB (*k* = 1) and UKDB (*k* = 2) in terms of zero-one loss.

Win/Draw/Loss	ETAN	UKDB (*k* = 2)
UKDB (*k* = 1)	6/2/2	
UKDB (*k* = 2)	7/1/2	6/2/2

## Appendix A

Table A1 shows the detailed results of zero-one loss for KDB (k=1), KDB (k=2), UKDB (k=1) and UKDB (k=2) on 10 datasets (described in Table 4). Table A2 shows the detailed results of zero-one loss for UKDB (k=2) with {MI, CMI} and {PMI, PCMI} on 10 datasets (described in Table 4). Table A3, Table A4, Table A5 show the detailed experimental results of average zero-one loss, bias and variance for NB, TAN, KDB and UKDB (k=2) on 10 datasets (described in Table 4), respectively. Table A6 shows the detailed zero-one loss results of TAN^e^, KDB^e^ and UKDB. In addition, Table A7 shows the detailed zero-one loss results of ETAN, UKDB (k=1) and UKDB (k=2).

**Table A1:** Detailed zero-one loss results of KDB (*k* = 1), KDB (*k* = 2), UKDB (*k* = 1) and UKDB (*k* = 2). The lowest results from all these BNCs are highlighted in bold.

Dataset	KDB (*k* = 1)	KDB (*k* = 2)	UKDB (*k* = 1)	UKDB (*k* = 2)
Adult	0.1758 ± 0.0041	0.1852 ± 0.0036	0.1676 ± 0.0035	**0.1537 ± 0.0045**
Census-Income	0.0736 ± 0.0020	0.0767 ± 0.0019	0.0740 ± 0.0037	**0.0689 ± 0.0022**
Dis	0.0186 ± 0.0047	0.0196 ± 0.0047	0.0141 ± 0.0027	**0.0177 ± 0.0047**
Hypothyroid	0.0127 ± 0.0049	0.0124 ± 0.0043	0.0121 ± 0.0062	**0.0112 ± 0.0065**
Chess	0.0998 ± 0.0023	0.0491 ± 0.0039	0.0644 ± 0.0023	**0.0414 ± 0.0061**
MAGIC	0.2042 ± 0.0105	0.2139 ± 0.0110	0.2021 ± 0.0081	**0.1987 ± 0.0101**
Mushroom	**0.0007 ± 0.0007**	0.0007 ± 0.0009	**0.0007 ± 0.0012**	0.0009 ± 0.0004
Musk	0.0713 ± 0.0157	0.0684 ± 0.0166	0.0706 ± 0.0197	**0.0654 ± 0.0167**
Sick	**0.0241 ± 0.0071**	0.0264 ± 0.0070	0.0266 ± 0.0062	0.0262 ± 0.0067
Spambase	0.0865 ± 0.0119	0.0742 ± 0.0142	0.0810 ± 0.0126	**0.0732 ± 0.0144**

**Table A2:** Detailed zero-one loss results of UKDB (*k* = 2) with {MI, CMI} and {PMI, PCMI}. The lowest results from all these BNCs are highlighted in bold.

Dataset	UKDB (*k* = 2) with {MI, CMI}	UKDB (*k* = 2) with {PMI, PCMI}
Adult	0.1537 ± 0.0045	0.1569 ± 0.0039
Census-Income	**0.0689 ± 0.0022**	0.0729 ± 0.0019
Dis	0.0177 ± 0.0047	0.0181 ± 0.0047
Hypothyroid	0.0112 ± 0.0065	0.0116 ± 0.0047
Chess	**0.0414 ± 0.0061**	0.0438 ± 0.0028
Magic	**0.1987 ± 0.0101**	0.2872 ± 0.0107
Mushroom	**0.0009 ± 0.0004**	0.0010 ± 0.0004
Musk	**0.0654 ± 0.0167**	0.0701 ± 0.0360
Sick	0.0262 ± 0.0067	0.0267 ± 0.0070
Spambase	0.0732 ± .00144	0.0743 ± 0.0126

**Table A3:** Experimental results of average zero-one loss for 10-cross validation. The lowest results from all these BNCs are highlighted in bold.

Dataset	NB	TAN	KDB (*k* = 2)	UKDB (*k* = 2)
Adult	0.1840 ± 0.0041	0.1765 ± 0.0039	0.1852 ± 0.0036	**0.1537 ± 0.0045**
Census-Income	0.1739 ± 0.0022	0.0736 ± 0.0022	0.0767 ± 0.0019	**0.0689 ± 0.0022**
Dis	0.0251 ± 0.0077	0.0197 ± 0.0054	0.0196 ± 0.0047	**0.0177 ± 0.0047**
Hypothyroid	0.0144 ± 0.0043	0.0128 ± 0.0048	0.0124 ± 0.0043	**0.0112 ± 0.0065**
Chess	0.1354 ± 0.0051	0.0853 ± 0.0092	0.0491 ± 0.0039	**0.0414 ± 0.0061**
MAGIC	0.2396 ± 0.0069	0.2149 ± 0.0098	0.2139 ± 0.0110	**0.1987 ± 0.0101**
Mushroom	0.0480 ± 0.0036	0.0008 ± 0.0004	**0.0007 ± 0.0009**	0.0009 ± 0.0004
Musk	0.1222 ± 0.0696	0.0890 ± 0.0132	0.0684 ± 0.0166	**0.0654 ± 0.0167**
Sick	0.0290 ± 0.0058	0.0296 ± 0.0061	0.0264 ± 0.0070	**0.0262 ± 0.0067**
Spambase	0.1069 ± 0.0127	0.0827 ± 0.0100	0.0742 ± 0.0142	**0.0732 ± 0.0144**

**Table A4:** Experimental results of average bias for 10-cross validation. The lowest results from all these BNCs are highlighted in bold.

Dataset	NB	TAN	KDB (*k* = 2)	UKDB (*k* = 2)
Adult	0.1649	0.1312	0.1220	**0.1127**
Census-Income	0.2303	0.0544	0.0421	**0.0396**
Dis	**0.0165**	0.0193	0.0191	0.0190
Hypothyroid	0.0116	0.0104	0.0096	**0.0083**
Chess	0.1107	0.0702	0.0417	**0.0401**
MAGIC	0.2111	0.1252	0.1241	**0.1203**
Mushroom	0.0237	**0.0001**	**0.0001**	**0.0001**
Musk	0.1847	0.1560	**0.1535**	0.1582
Sick	0.0246	0.0207	0.0198	**0.0193**
Spambase	0.0929	0.0570	**0.0497**	0.0532

**Table A5:** Experimental results of average variance for 10-cross validation. The lowest results from all these BNCs are highlighted in bold.

Dataset	NB	TAN	KDB (*k* = 2)	UKDB (*k* = 2)
Adult	**0.0069**	0.0165	0.0285	0.0164
Census-Income	**0.0052**	0.0101	0.0110	0.0060
Dis	**0.0001**	0.0005	0.0011	0.0002
Hypothyroid	**0.0017**	0.0021	0.0024	0.0026
Chess	0.0186	0.0102	0.0111	**0.0096**
MAGIC	**0.0174**	0.0490	0.0491	0.0457
Mushroom	0.0043	**0.0002**	**0.0002**	**0.0002**
Musk	**0.1108**	0.1180	0.1320	0.1169
Sick	0.0047	0.0051	**0.0043**	0.0048
Spambase	**0.0092**	0.0158	0.0214	0.0152

**Table A6:** Detailed zero-one loss results of TAN^*e*^, KDB^*e*^ (*k* = 2) and UKDB (*k* = 2). The lowest results from all these BNCs are highlighted in bold.

Dataset	TAN^*e*^	KDB^*e*^ (*k* = 2)	UKDB (*k* = 2)
Adult	0.1554 ± 0.0037	0.1601 ± 0.0039	**0.1537 ± 0.0045**
Census-Income	0.0784 ± 0.0030	0.0729 ± 0.0027	**0.0689 ± 0.0022**
Dis	0.0180 ± 0.0041	0.0185 ± 0.0034	**0.0177 ± 0.0047**
Hypothyroid	0.0120 ± 0.0055	0.0120 ± 0.0056	**0.0112 ± 0.0065**
Chess	0.0701 ± 0.0058	0.0463 ± 0.0089	**0.0414 ± 0.0061**
Magic	0.2177 ± 0.0090	0.2157 ± 0.0097	**0.1987 ± 0.0101**
Mushroom	**0.0008 ± 0.0008**	0.0010 ± 0.0011	0.0009 ± 0.0004
Musk	0.0828 ± 0.0165	0.0749 ± 0.0190	**0.0654 ± 0.0167**
Sick	0.0320 ± 0.0062	0.0272 ± 0.0071	**0.0262 ± 0.0067**
Spambase	0.0849 ± 0.0113	0.0755 ± 0.0132	**0.0732 ± 0.0144**

**Table A7:** Detailed zero-one loss results of ETAN, UKDB (*k* = 1) and UKDB (*k* = 2). The lowest results from all these BNCs are highlighted in bold.

Dataset	ETAN	UKDB (*k* = 1)	UKDB (*k* = 2)
Abalone	**0.1180 ± 0.0043**	0.1676 ± 0.0035	0.1537 ± 0.0045
Census-Income	0.0733 ± 0.0017	0.0740 ± 0.0037	**0.0689 ± 0.0022**
Dis	0.0194 ± 0.0040	**0.0141 ± 0.0027**	0.0177 ± 0.0047
Hypothyroid	0.0113 ± 0.0049	0.0121 ± 0.0062	**0.0112 ± 0.0065**
Chess	0.0746 ± 0.0054	0.0644 ± 0.0023	**0.0414 ± 0.0061**
Magic	0.2157 ± 0.0112	0.2021 ± 0.0081	**0.1987 ± 0.0101**
Mushroom	0.0008 ± 0.0009	**0.0007 ± 0.0012**	0.0009 ± 0.0004
Musk	0.0789 ± 0.0182	0.0706 ± 0.0197	**0.0654 ± 0.0167**
Sick	0.0281 ± 0.0080	0.0266 ± 0.0062	**0.0262 ± 0.0067**
Spambase	0.0838 ± 0.0137	0.0810 ± 0.0126	**0.0732 ± 0.0144**
